# Readiness to Embrace Artificial Intelligence Among Medical Doctors and Students: Questionnaire-Based Study

**DOI:** 10.2196/34973

**Published:** 2022-04-12

**Authors:** Thomas Boillat, Faisal A Nawaz, Homero Rivas

**Affiliations:** 1 Design Lab, College of Medicine Mohammed Bin Rashid University of Medicine and Health Sciences Dubai United Arab Emirates; 2 College of Medicine Mohammed Bin Rashid University of Medicine and Health Sciences Dubai United Arab Emirates

**Keywords:** artificial intelligence in medicine, health care, questionnaire, medical doctors, medical students

## Abstract

**Background:**

Similar to understanding how blood pressure is measured by a sphygmomanometer, physicians will soon have to understand how an artificial intelligence–based application has come to the conclusion that a patient has hypertension, diabetes, or cancer. Although there are an increasing number of use cases where artificial intelligence is or can be applied to improve medical outcomes, the extent to which medical doctors and students are ready to work and leverage this paradigm is unclear.

**Objective:**

This research aims to capture medical students’ and doctors’ level of familiarity toward artificial intelligence in medicine as well as their challenges, barriers, and potential risks linked to the democratization of this new paradigm.

**Methods:**

A web-based questionnaire comprising five dimensions—demographics, concepts and definitions, training and education, implementation, and risks—was systematically designed from a literature search. It was completed by 207 participants in total, of which 105 (50.7%) medical doctors and 102 (49.3%) medical students trained in all continents, with most of them in Europe, the Middle East, Asia, and North America.

**Results:**

The results revealed no significant difference in the familiarity of artificial intelligence between medical doctors and students (*P*=.91), except that medical students perceived artificial intelligence in medicine to lead to higher risks for patients and the field of medicine in general (*P*<.001). We also identified a rather low level of familiarity with artificial intelligence (medical students=2.11/5; medical doctors=2.06/5) as well as a low attendance to education or training. Only 2.9% (3/105) of medical doctors attended a course on artificial intelligence within the previous year, compared with 9.8% (10/102) of medical students. The complexity of the field of medicine was considered one of the biggest challenges (medical doctors=3.5/5; medical students=3.8/5), whereas the reduction of physicians’ skills was the most important risk (medical doctors=3.3; medical students=3.6; *P*=.03).

**Conclusions:**

The question is not whether artificial intelligence will be used in medicine, but when it will become a standard practice for optimizing health care. The low level of familiarity with artificial intelligence identified in this study calls for the implementation of specific education and training in medical schools and hospitals to ensure that medical professionals can leverage this new paradigm and improve health outcomes.

## Introduction

### Background

Both public news and scientific articles widely argue that artificial intelligence will eventually disrupt medicine and the way physicians and medical professionals will be practicing in the future [1,2]. There has been impactful research that demonstrates the potential of artificial intelligence in medicine (AIM), for instance, to classify images such as x-rays [3]. Artificial intelligence is being evaluated not only for image processing and analysis but also for prognosis [4-6], treatment [7-9], and patient monitoring [10,11] among other uses. In addition, artificial intelligence algorithms have also been implemented in many consumer health products such as wearables and mobile devices [12]. From a medical perspective, it means that artificial intelligence–based algorithms are already giving recommendations to both patients and physicians and taking decisions on their behalf. It is therefore critical that physicians understand how this approach works and for software vendors and hospitals to identify what physician needs are to facilitate its implementation. So far, the evidence has not been very reassuring. When asked, “How familiar are you with artificial intelligence?” only 6% (out of a sample of 669 participants) of physicians and physicians in training in Seoul answered positively [13]. In another recent study, French medical experts reported that artificial intelligence is a “fuzzy notion” [2]. To evaluate the amount of empirical evidence collected regarding medical doctors’ (MDs) and medical students’ (MSs) level of understanding toward AIM, we conducted a systematic literature research. Of the 96 articles collected from Scopus, we identified only 9 (9%) studies (Multimedia Appendix 1 [2,11,13-19]) that surveyed medical professionals, the other ones being either out of scope or literature reviews. From existing empirical research, we identified the following. First, most studies surveyed medical professionals from either 1 university or 1 country. Second, one-third of the studies focused on the use of artificial intelligence in radiology. Third, none of the existing studies aimed to assess the level of understanding toward AIM.

### Objectives

Owing to the importance of the topic, with this research, we intend to close this gap by surveying MDs and MSs from around the world on AIM topics that are the most discussed in the current literature. On the basis of the literature search, our questionnaire comprises the following sections: (1) the level of familiarity with AIM, (2) education and training related to AIM, (3) challenges and barriers linked to the implementation of artificial intelligence in clinical settings, and (4) risks linked to AIM.

## Methods

Data were collected by means of a web-based questionnaire built following the guidelines developed by Burgess [20] as well as the 7-step process by Fowler [21].

### Step 1—Define Your Research Aims

On the basis of the existing literature, we identified limited empirical data regarding MSs’ and physicians’ level of understanding toward AIM, their participation to AIM education, and challenges and barriers related to AIM implementation as well as potential risks linked to the democratization of AIM in clinical settings.

### Step 2—Identify the Population and Sample

We were particularly interested in comparing MSs as a population (eg, aged 18-25 years) who has grown with technology and practicing physicians (eg, aged 30-60 years) who have clinical experience but might have been less exposed to technology. Mindful that the place of study and employment has a direct link with the knowledge and expertise that one acquires, we targeted the 6 continents to have a broad representation of the population under investigation. Participants were recruited by means of individual emails and posts from the authors’ (TB, FAN, and HR) LinkedIn and Twitter profile feeds. This technique was used to avoid having participants from the same medical schools or hospitals, potentially having received the same education or training and thus creating biases in the data. To detect potential biases, we used the graduation year and name of the university to identify participants from the same cohort. When we found participants from the same school and graduation year, we randomly chose 5 of them. On the basis of the goal of this research, which investigates differences between two independent populations, MDs and MSs, our data sample should not be smaller than 176. This number was calculated based on a medium effect size (Cohen *d*) of 0.5, referring to limited existing empirical evidence [22]. Power was set to 0.95 with an allocation ratio of 1.

### Step 3—Decide How to Collect Replies

Data were centralized in the university’s platform after being collected by means of web-based questionnaire (Microsoft Forms).

### Step 4—Design Your Questionnaire

From our review of previous work, we identified that none of the existing questionnaires were built following a systematic approach. In most studies, AIM factors were chosen based on research motivation. To systematically cover the most relevant AIM factors, we conducted a systematic literature search following the PRISMA (Preferred Reporting Items for Systematic Reviews and Meta-Analyses) guidelines [23]. To this end, we performed a title and keyword search on the Scopus database using the keywords *“artificial intelligence”* AND *medicine* OR *“machine learning”* AND *medicine* as well as *“artificial intelligence”* AND *healthcare* OR *“machine learning”* AND *healthcare*. We did not perform an abstract search because of the abundance of unrelated articles. The search resulted in 837 papers. After being reviewed by 2 independent researchers for consistency, 9.3% (78/837) of the studies were retained for our qualitative analysis; of the 78 studies, only 9 (12%) used questionnaires. In total, 244 sections and 405 subsections were extracted. The latter were clustered by 2 independent researchers based on their similarity. From the 11 clusters, we created four different groups: (1) concepts and definitions, (2) training and education, (3) implementation, and (4) risks. A 5-point Likert scale was used for most questions, and drop-down menus were used for questions requiring categorical answers. More specifically, questions in the concepts and definitions factor displayed the following scales: (1) I have never heard of it, (2) I have heard of it a few times, (3) I understand it, (4) I can potentially explain it, and (5) I can confidently explain it. We defined the AIM’s level of familiarity by calculating the mean across the different factors (questions 1.1-1.10 of Table 1). Questions in the training and education factor displayed the following scales: (1) strongly disagree, (2) disagree, (3) neutral, (4) agree, and (5) strongly agree. Questions in the implementation and risks factors displayed a scale similar to that in the training and education factors, but with an added option (0), I do not know. Finally, the clinical experience (only MDs) was derived from each age group as follows: 20 to 29 years=1, 30 to 39 years=2, 40 to 49 years=3, 50 to 59 years=4, and 60 to 69 years=5. The questionnaire can be accessed via Multimedia Appendix 2.

**Table 1:** Mean (SD) and *P* value for each factor.

Factors				MD^a^, mean (SD)		MS^b^, mean (SD)		*P* value
**1. Familiarity with AIM^c^**								
	1.1	AI^d^	3.1 (1.0)		3.3 (1.0)		.24	
	1.2	ML^e^	2.7 (1.1)		2.8 (1.2)		.46	
	1.3	Supervised ML	2.0 (1.2)		2.1 (1.2)		.77	
	1.4	Unsupervised ML	1.9 (1.1)		2.0 (1.2)		.87	
	1.5	Deep learning	2.2 (1.1)		2.4 (1.2)		.18	
	1.6	Neural networks	2.2 (1.1)		2.5 (1.2)		.14	
	1.7	Fuzzy logic	1.6 (0.9)		1.5 (0.9)		.48	
	1.8	Support vector machine	1.5 (0.9)		1.4 (0.8)		.37	
	1.9	Overfitting or underfitting	1.6 (1.1)		1.5 (1.0)		.83	
	1.10	Feature selection	1.7 (1.1)		1.8 (1.1)		.64	
**2. Education and training**								
	2.1	Last time an AIM course was attended	1.4 (1.0)		1.98 (1.5)		.006^f^	
	2.2	Better understand the main concepts of artificial intelligence	4.0 (1.0)		4.3 (0.8)		.08	
	2.3	Explore the opportunities offered by artificial intelligence in general	4.1 (1.1)		4.2 (0.9)		.36	
	2.4	Explore the opportunities offered by AIM and your field	4.1 (1.1)		4.3 (0.8)		.14	
	2.5	Know more of existing commercial solutions	3.8 (1.1)		4.0 (0.9)		.23	
	2.6	Create my own artificial intelligence algorithm or applications	3.8 (1.1)		3.7 (1.1)		.40	
**3. Challenges to AIM’s implementation**								
	3.1	Outcomes of artificial intelligence algorithms are difficult to trace or understand (the black box syndrome)	2.8 (1.7)		2.9 (1.7)		.67	
	3.2	The complexity of the field of medicine	3.5 (1.5)		3.8 (1.3)		.12	
	4.3	The availability of high-quality data samples	3.7 (1.4)		3.3 (1.7)		.75	
	3.4	The artificial intelligence’s level of autonomy (what artificial intelligence should and should not do)	3.7 (1.4)		3.7 (1.4)		.96	
	3.5	The costs associated with the implementation of artificial intelligence	3.4 (1.6)		3.75 (1.4)		.16	
	3.6	Data privacy or confidentiality	3.7 (1.5)		3.7 (1.5)		.79	
**4. Barriers to AIM’s implementation**								
	4.1	The availability of comparison studies	3.7 (1.4)		3.3 (1.7)		.13	
	4.2	The safe use of artificial intelligence	3.9 (1.3)		4.0 (1.4)		.93	
	4.3	Build trust between humans and artificial intelligence	3.7 (1.5)		3.7 (1.5)		.88	
	4.4	Availability of regulations and legislation	3.7 (1.6)		3.8 (1.6)		.75	
	4.5	The top management’s level of understanding	3.8 (1.5)		3.6 (1.6)		.45	
**5. Risks linked to AIM’s implementation**								
	5.1	Dehumanization of health care	3.3 (1.2)		3.5 (1.1)		.12	
	5.2	Reduction in physicians’ skills (eg, physicians might execute fewer types of tasks)	3.3 (1.2)		3.6 (1.0)		.03^f^	
	5.3	Artificial intelligence will eventually harm patients	2.3 (0.9)		2.8 (1.0)		<.001^f^	
	5.4	Physicians may become redundant	2.6 (1.1)		3.0 (1.1)		.008^f^	

^a^MD: medical doctor.

^b^MS: medical student.

^c^AIM: artificial intelligence in medicine.

^d^AI: artificial intelligence.

^e^ML: machine learning.

^f^Significant difference.

### Step 5—Run a Pilot Survey

The questionnaire was completed by 15 MSs and 17 physicians from six different regions (Asia, Oceania, North America, the Middle East, Europe, and Eastern Europe). Cronbach α coefficient values of internal reliability reached .85, above the accepted .70 threshold [24]. When unpacked, the four quantitative parts (ie, concepts and definitions, training and education, implementation, and risks) respectively reached the following coefficient of internal reliability: (1) .91, (2) .94, (3) .81, and (4) .81. We used principal component analysis to examine the factor structure of the questionnaire. Kaiser-Meyer-Olkin (KMO) factor adequacy showed no correlation across the four factors (concepts and definitions, training and education, implementation, and risks). However, we did find the following correlations: (1) KMO=0.72, (2) KMO=0.75, (3) KMO=0.58, and (4) KMO=0.62. Following Kaiser and Rice [25], values above 0.5 are considered acceptable.

### Step 6—Conduct the Main Survey

Participants used the link displayed in LinkedIn and Twitter posts to open the questionnaire. The landing page displayed the consent form including the objective and nature of the research, the risks and benefits, compensation and costs, confidentiality, participation (including rights to withdraw), contact information, and instruction. Only after choosing “I accept,” were the participants redirected to the questionnaire. The recruitment and questionnaire were open from August to December 2020.

### Step 7—Data Analysis

We used descriptive statistics to describe and compare the demographics as well as the distributions of MDs and MSs within the four different factors (concepts and definitions, training and education, implementation, and risks). We then tested the descriptive statistics between MDs and MSs for significant differences using unpaired 2-tailed *t* tests (95% CI). We also built a linear regression model to explore factors associated with the risks brought by AIM (the risks factor). *P*<.05 is considered statistically significant. The outlined methods were carried out in accordance with relevant guidelines and regulations. We relied on the *required* functionality of our survey tool to ensure that participants did not miss any questions. As a result, no missing data were observed.

### Ethics Approval

This study was approved by the Mohammed Rashid University of Medicine and Health Sciences’ Institutional Review Board Committee under MBRU-IRB-2020-024, and informed consent was obtained from all participants. The CHERRIES (Checklist for Reporting the Results of Internet E-Surveys) for the distributed survey is included as Multimedia Appendix 3 [26].

## Results

### Demographics

A total of 207 completed questionnaires were received. Among these 207 questionnaires, 105 (50.7%) were practicing physicians holding a medical degree and 102 (49.3%) were MSs. The repartition between men and women is somewhat even, as shown in Table 2. Although most of the participants were based in the Middle East (100/207, 48.3%), only 24.3% (51/207) of them were trained or are receiving their medical education in the Middle East (the list of institutions is available in Multimedia Appendix 4). Europe and Asia followed, with 24.8% (52/207) of the participants having received or are receiving their education in Europe and 15.8% (33/207) in Asia, whereas 17.4% (36/207) of the participants were based in Europe and 18.8% (39/207) in Asia. The distribution of participants can be found in Multimedia Appendix 4. The average time to complete the questionnaire was 12 minutes.

**Table 2:** Participants’ demographics (N=207).

Demographics		MD^a^, n (%)	MS^b^, n (%)	Total, n (%)
Participants		105 (50.1)	102 (49.9)	207 (100.0)
**Sex**				
	Men	62 (59.1)	43 (40.9)	105 (50.1)
	Women	43 (42.1)	59 (57.9)	102 (49.9)
**Age (years)**				
	<20	0 (0)	19 (18.6)	19 (9.2)
	20-29	18 (17.1)	82 (80.4)	100 (48.3)
	30-39	27 (25.7)	1 (0.9)	28 (13.3)
	40-49	26 (24.8)	0 (0)	26 (12.6)
	50-59	26 (24.8)	0 (0)	26 (12.6)
	60-69	8 (7.6)	0 (0)	8 (3.9)
	>70	0 (0)	0 (0)	0 (0)
**Where the highest medical degree was obtained**				
	Asia	14 (13.3)	19 (18.6)	33 (15.9)
	Africa	4 (3.8)	3 (2.9)	7 (3.4)
	Central America	0 (0)	0 (0)	0 (0)
	North America	11 (10.5)	10 (9.8)	21 (10.1)
	South America	1 (0.9)	0 (0)	1 (0.5)
	Europe	29 (27.6)	23 (22.6)	52 (25.1)
	Eastern Europe	0 (0)	1 (0.9)	1 (0.5)
	Middle East	9 (8.6)	42 (41.2)	51 (24.6)
	Oceania	1 (0.9)	2 (1.9)	3 (1.4)
**Where the participants are based**				
	Asia	15 (14.3)	24 (23.5)	39 (18.8)
	Africa	1 (0.9)	4 (3.9)	5 (2.4)
	Central America	2 (1.9)	0 (0)	2 (0.9)
	North America	11 (10)	9 (8)	20 (9)
	South America	0 (0)	0 (0)	0 (0)
	Europe	15 (14.1)	21 (20.6)	36 (17.4)
	Eastern Europe	0 (0)	1 (0.9)	1 (0.5)
	Middle East	59 (56.2)	41 (40.2)	100 (48.3)
	Oceania	2 (1.9)	2 (1.9)	4 (1.9)

^a^MD: medical doctor.

^b^MS: medical student.

### Main Outcomes

As shown in Table 1, artificial intelligence (1.1) is the only concept that most participants understand with a mean of 3.27 (SD 1) for MSs and 3.11 (SD 1) for MDs. It is followed by machine learning (1.2), neural networks (1.6), and deep learning (1.5). Supervised and unsupervised machine learning (1.3 and 1.4), which are two concepts widely used in medicine, did not score very high.

**Figure 1:**
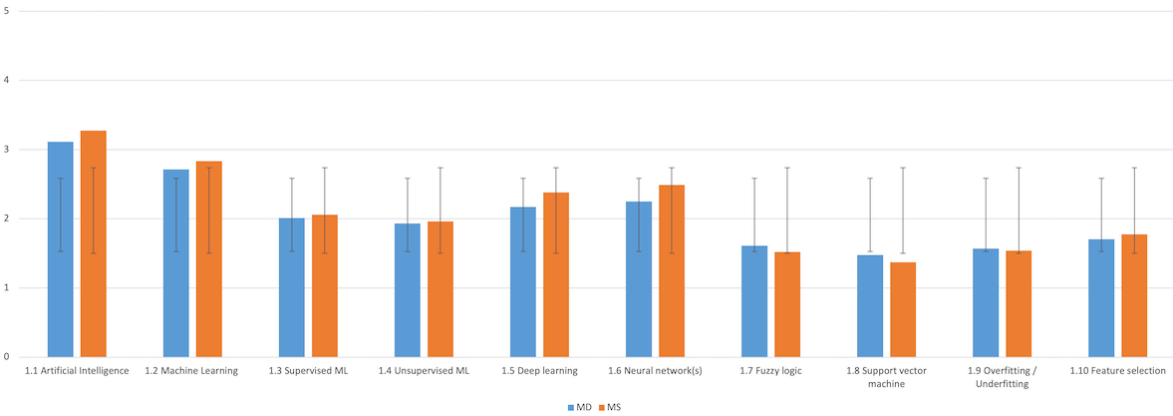
Familiarity with artificial intelligence in medicine (AIM)—comparison between medical doctor (MD) and medical student (MS; y-axis: means and SDs). ML: machine learning.

The concept of overfitting and underfitting (1.9), which is one of the core principles in artificial intelligence, obtained among the lowest scores. In addition to questions 1.7, 1.8, and 1.9, MSs showed a better level of understanding than MDs as displayed in Figure 1. However, statistical comparisons between the 2 populations revealed no significant difference across the artificial intelligence concepts, as shown in Table 1.

**Figure 2:**
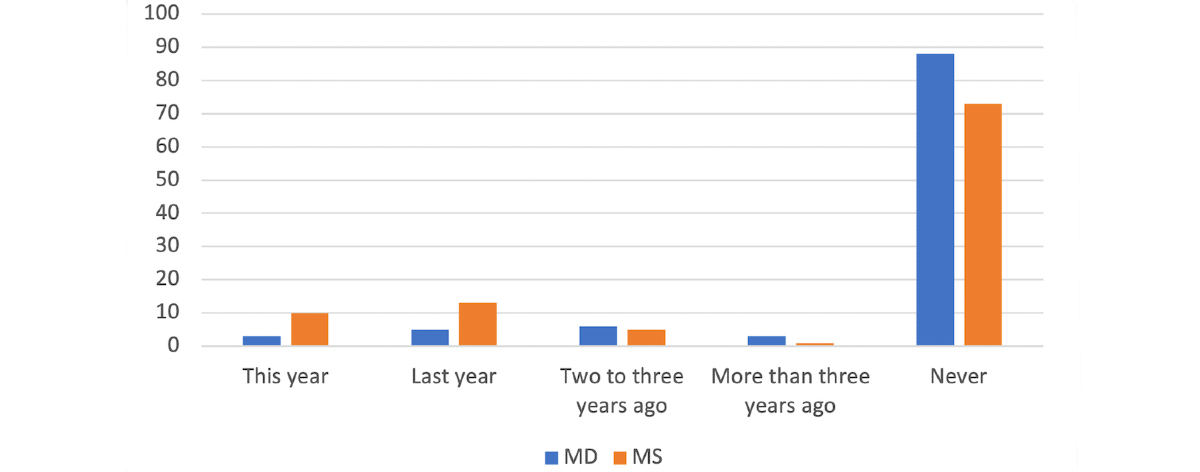
Last time that medical doctor (MD) and medical student (MS) attended a course on artificial intelligence in medicine (AIM; y-axis: percentages).

We asked, “When was the last time you attended a course on AIM?” (2.1), a large majority of both MDs and MSs have never attended a course on AIM, whereas slightly more MSs have done so this year (ie, in 2020) or last year (Figure 2). Tests of statistical significance showed a difference between the 2 populations as shown in Table 1.

**Figure 3:**
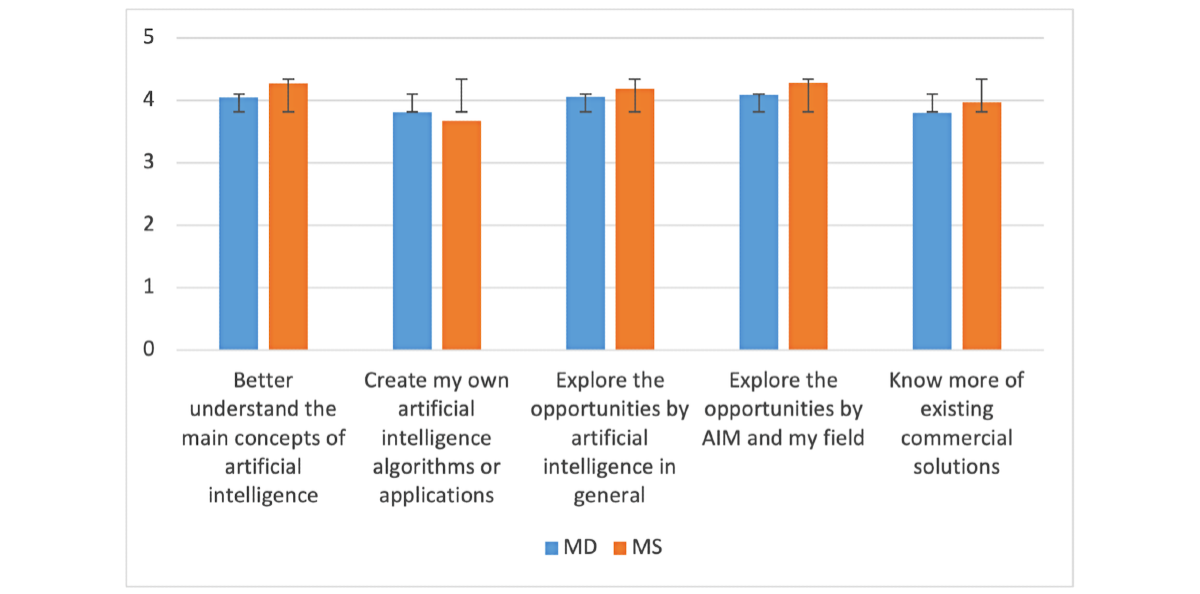
Reasons to attend a course on artificial intelligence in medicine (AIM)—comparison between medical doctor (MD) and medical student (MS; y-axis: means and SDs).

For both MDs and MSs, the priority is to further explore opportunities offered by artificial intelligence in their own field and in general and to better understand the main concept of artificial intelligence. Despite MDs having clinical expertise and, thus, a better idea of potential opportunity, there was no significant difference with MSs. However, the fact that MDs are more eager to learn how to create their own artificial intelligence algorithms or applications might confirm that they see more clinical potential than MSs, as displayed in Figure 3.

**Figure 4:**
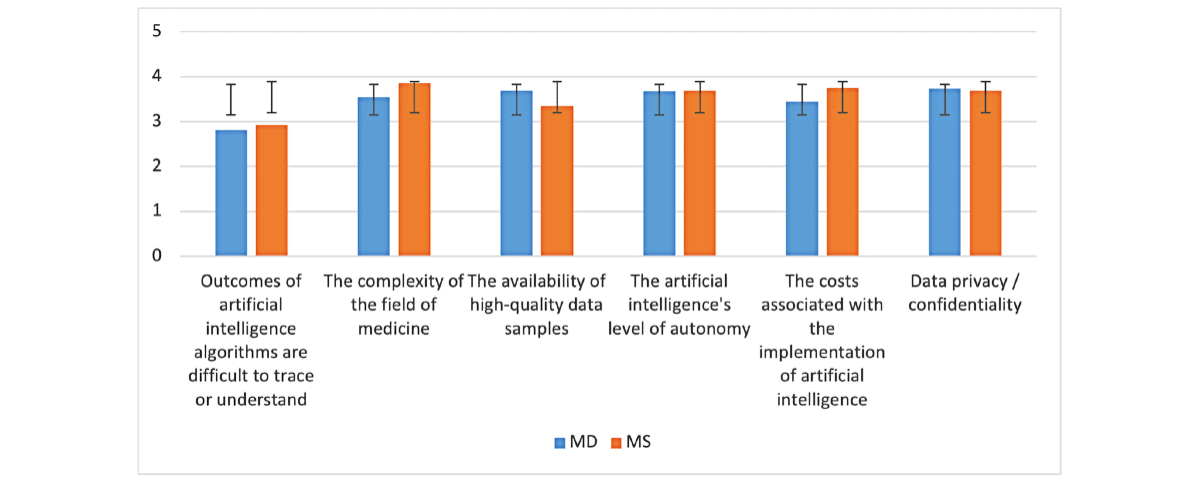
Challenges to artificial intelligence in medicine’s (AIM) implementation—comparison between medical doctor (MD) and medical student (MS; y-axis: means and SDs).

From an MD perspective, challenges linked to data privacy and confidentiality are the biggest challenges, followed by the availability of high-quality data samples and the artificial intelligence’s level of autonomy, as shown in Figure 4. From an MS viewpoint, the complexity of the field of medicine is the biggest challenge, which could be explained by their limited expertise and clinical exposure. This is followed by the cost associated with the implementation of artificial intelligence as well as the artificial intelligence’s level of autonomy and challenges related to data privacy and confidentiality. Challenges caused by the black box syndrome drew the least attention. There was no statistical difference between MDs and MSs across the different challenges.

**Figure 5:**
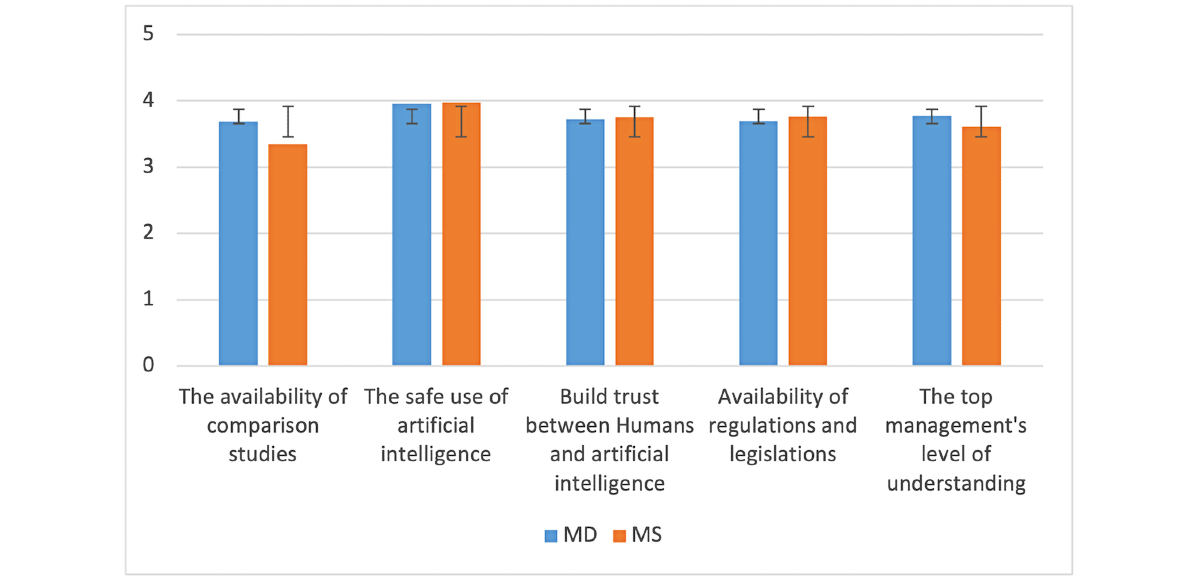
Barriers to artificial intelligence in medicine’s (AIM) implementation—comparison between medical doctor (MD) and medical student (MS; y-axis: means and SDs).

In addition to challenges linked to the implementation of AIM, we also identified in the literature some barriers that can prevent the implementation of AIM, as displayed in Figure 5. From both the MD and MS perspectives, the safe use of artificial intelligence is the most important, followed by the availability of regulations and legislation as well as trust that must be built between human and artificial intelligence. The top management’s level of understanding is the only one that MDs rated higher than MSs. For these factors, too, we did not find a significant difference between MDs and MSs.

**Figure 6:**
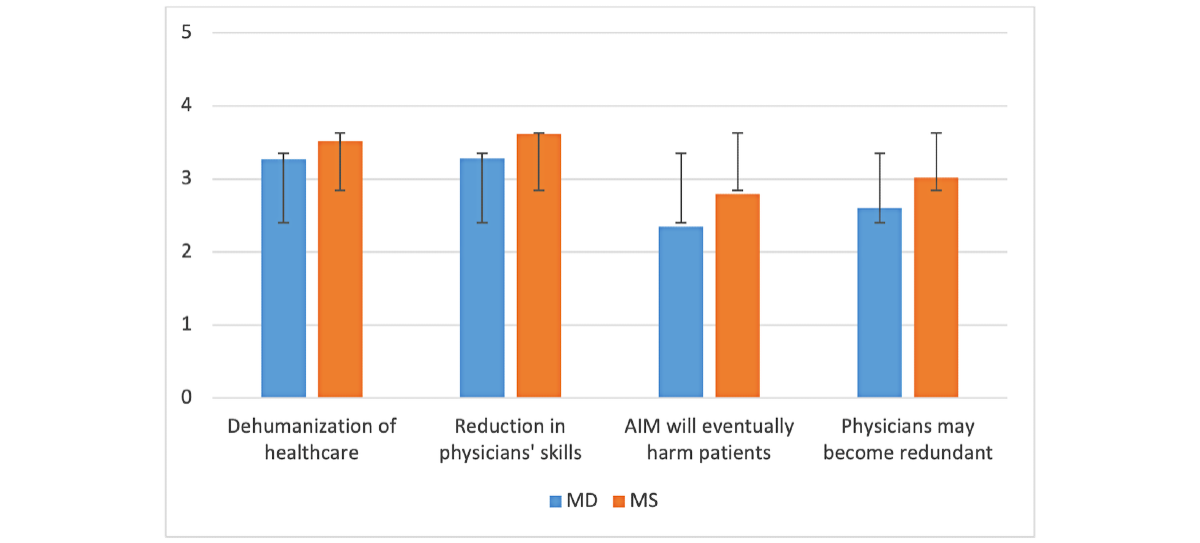
Risks linked to artificial intelligence in medicine’s (AIM) implementation—comparison between medical doctor (MD) and medical student (MS; y-axis: means and SDs).

Among the risks linked to the use of artificial intelligence in clinical settings, the potential reduction in physicians’ skills was rated the highest by both MDs and MSs, as shown in Figure 6. With a score of 3.29 and 3.62, given by MDs and MSs, respectively, the test for statistical difference was significantly positive. The second highest score went to the risk linked to the dehumanization of health care with 3.27 for MDs and 3.52 for MSs. MSs are also more concerned than MDs that physicians may become redundant and that artificial intelligence will eventually harm patients, and both showed statistical differences between the 2 groups (*P*=.008 and *P*<.001, respectively).

**Figure 7:**
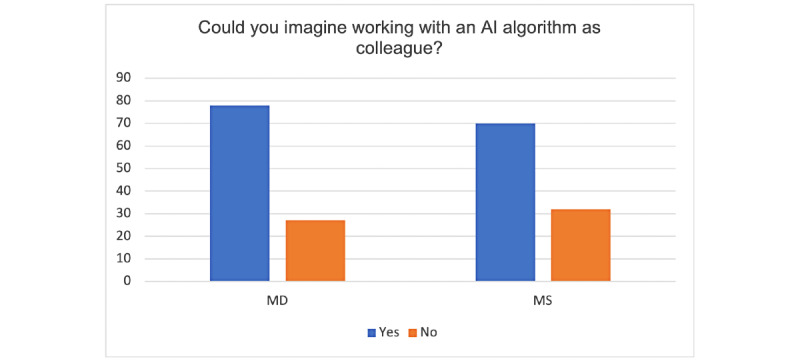
Working with an artificial intelligence (AI) algorithm—results (y-axis: percentages).

The questionnaire ended with the following question: Can you imagine working with an artificial intelligence algorithm as a colleague? Most participants answered “yes” as shown in Figure 7.

We were interested to know more about the underlying reasons behind this choice and thus asked the participants to motivate their answer. Textbox 1 presents the extract of the collected responses.

We were interested in investigating the relationships between the level of familiarity with AIM, clinical experience, and the perception of risks. As shown in Table 3, there are significant negative correlations between the level of familiarity with AIM and the risk of dehumanization of health care, reduction in physicians’ skills, and risk that physicians may become redundant. In other words, the more MDs and MSs know about AIM, the less they perceive these factors as risks. No significant difference was identified between the level of AIM familiarity and the risk to eventually harm patients. Similarly, there are significant negative correlations between clinical experience and the risk that artificial intelligence will eventually harm patients and that physicians become redundant. No significant difference was identified with the risk that artificial intelligence will dehumanize health care or reduce physicians’ skills.

**Why could you (not) work with artificial intelligence as a colleague? Answers from participants.**

**Yes**

- “That is the future - safer, secure, less emotionally driven, more reliable.”
- “Ease the work, accurate diagnosis, improve patient care and reduce workload.”
- “It would be very efficient and helpful as information will be processed and delivered instantly with less room for error.”
- “Because an artificial intelligence will help in reducing the human errors such as near misses or misdiagnosis. It will learn the more it sees and will adapt to the patient presentation just as we medical students do.”
- “The speed of development is exponential and the current status is quite impressive.”

**No**

- “Physicians are being undermined and eventually replaced by mid-level providers and artificial intelligence.”
- “Because although we already deal with ‘algorithms’ that have the potential to become artificial intelligence algorithms in our academic learning. I have not encountered many physicians who adopt that way of linear thinking in their practice. To them, intuition plays a bigger role in clinical judgment.”
- “I think artificial intelligence should only be bossed around and not seen as a colleague who can think by himself because artificial intelligence cannot have moral or emotional values from itself but from a human boss who manages or controls it.”
- “Artificial intelligence is OK for hypothesis generation, e.g., suggesting rare diagnoses which may be missed, but cannot replace the dynamic interaction with knowledgeable colleagues.”
- “I don’t think artificial intelligence will be able to communicate like my colleagues in my lifetime.”

**Table 3:** Factors associated with AIM^a^ risks (significance level *P*>.05).

Associated factors			Estimate (SE; SD)		*t* test (*df*)		*P* value
**Dehumanization of health care**							
	Familiarity with AIM	−0.28393 (0.09757; 1.146)		−2.91 (205)		.004^b^	
	Clinical experience	−0.08585 (0.0608; 1.164)		−1.415 (205)		.16	
**Reduction in physicians’ skills**							
	Familiarity with AIM	−0.27568 (0.09252; 1.087)		−2.98 (205)		.003^b^	
	Clinical experience	−0.10175 (0.05743; 1.102)		−1.772 (205)		.08	
**Artificial intelligence will eventually harm patients**							
	Familiarity with AIM	−0.0949 (0.08102; 0.952)		−1.171 (205)		.24	
	Clinical experience	−0.12819 (0.04897; 0.940)		−2.618 (205)		.009^b^	
**Physicians may become redundant**							
	Familiarity with AIM	−0.21163 (0.09575; 1.125)		−2.21 (205)		.28^b^	
	Clinical experience	−0.14515 (0.05846; 1.122)		−2.483 (205)		.01^b^	

^a^AIM: artificial intelligence in medicine.

^b^Significant difference.

## Discussion

### Principal Findings

This research focused on assessing the level of understanding of AIM of MDs and MSs by means of a web-based questionnaire. It aims to complement the limited number of empirical studies on this key topic. When asked about artificial intelligence fundamentals, the participants provided somehow inconsistent answers. If most MDs and MSs understand artificial intelligence as a concept, it is unclear why they have only heard of overfitting and underfitting a few times, although these 2 concepts are key to understanding the outcomes of an artificial intelligence algorithm and their impact [27]. Similarly, the concepts of supervised and unsupervised algorithms did not reach a high level of familiarity for either MDs or MSs, whereas deep learning and neural networks, the 2 most used types of algorithms in supervised settings, received a higher score.

It was reassuring to find a strong positive correlation between deep learning (1.5) and overfitting or underfitting (1.9) as well as between neural networks (1.6) and overfitting or underfitting. This means that those who have a good level of understanding of deep learning and natural networks also have a good understanding of overfitting or underfitting. When analyzed at an aggregated level (questions 1.1–1.10), our results did not reveal any significant difference between MDs and MSs, which was unexpected because of the high level of curiosity of the younger population when it comes to technology and innovation. Globally, this low level of familiarity with artificial intelligence is not surprising when looking at the low number of MDs or MSs who had attended a course on artificial intelligence (Figure 2). Our analysis also showed that participants who attended a course on artificial intelligence have a statistically significant level of familiarity with artificial intelligence (*P*<.001). According to a recent study, a large majority of MSs argued that artificial intelligence should be part of medical training [17], although very few medical schools offer such programs [28].

When it came to the challenges linked to the implementation of AIM, we did not expect to observe a statistically significant difference between the 2 groups. We expected that clinical experience and an understanding of clinic organization would play a role in evaluating potential challenges. It can also be explained by the low level of artificial intelligence familiarity, which can limit MDs in understanding where AIM could bring new opportunities. It was also not expected that the *black box* syndrome would not be perceived as a bigger challenge (MDs=2.82; MSs=2.92). Such a lack of transparency is exactly what medicine does not want to see and has been identified as high risk by many scholars and practitioners [29-33]. These results also contradict the high importance that both MDs and MSs put in building trust between artificial intelligence and humans, which is very challenging owing to the lack of algorithms’ transparency. Both the MDs and MSs also showed concerns with the safe use of artificial intelligence and the existence of regulations and legislation. Some efforts are being made with, for instance, the *Proposed Regulatory Framework for Modifications to Artificial Intelligence/Machine Learning-Based Software as a Medical Device*. The draft of this document, published by the US Food and Drug Administration, seeks feedback from experts [34]. It was also very interesting to discover that MSs are genuinely more risk averse than MDs. They fear that artificial intelligence might reduce physicians’ skills, eventually harm patients, and make physicians redundant. These results can be partially explained by the correlation between risks and years of experience. As shown in Table 3, the more time spent in clinics, the lower the perceived risks caused by artificial intelligence. When analyzing the reasons why MDs and MSs could be willing to work with artificial intelligence as a colleague, it appears that the opportunities offered by artificial intelligence to improve patient care and reduce human errors are the most prevalent. Conversely, participants who said that they could not work with an artificial intelligence algorithm did not necessarily disagree with using artificial intelligence but rather with seeing artificial intelligence as a colleague, as they argued that technology can have neither the same emotion as humans nor the same way of thinking and interaction.

### Limitations

Some limitations with our research and its data sample in particular should be considered. First, our recruitment technique limited the reach of the questionnaire to participants as part of the network of the authors. Consequently, some regions of the world are underrepresented. In addition, it did not allow us to systematically calculate the response rate. Second, although the sample size was statistically sufficient for our research goals, it did not allow us to further investigate the differences across variables such as regions, age groups, education (eg, undergraduate programs vs postgraduate programs), or medical specializations.

### Comparison With Previous Work

This research differentiates itself from existing studies [8,17,35] through its approach and the diversity of its data sample. By building our questionnaire based on a literature search, we ensured that the most common AIM topics are included in our questionnaire. This approach is unique among similar studies that rather selected their AIM scope based on unknown criteria. As a result, our questionnaire is also more specific compared with existing research. For instance, in their study, Oh et al [13] asked, “Do you agree that you have good familiarity with artificial intelligence? [Strongly] agree, neutral, [strongly] disagree.” Instead, we decoupled the same question in 10 specific subtopics from machine learning to deep learning. In addition to a thinner abstraction level, it allowed us to identify some inconsistencies in some answers where some participants were supposedly able to confidently explain machine learning, but they had never heard of unsupervised algorithms, which is very unlikely. In addition, unlike most of existing work, we combined quantitative with qualitative data, which allowed us to know the *why*. When it comes to our data sample, its characteristics are also unique. Most specifically, our data sample is more diverse than those in existing research, with participants having studied (or studying) in 128 different universities across 6 continents. In contrast, in a study by Santos et al [17], the 263 answers were collected from 3 universities only. The likelihood that participants received the same education is rather high, bringing potential biases in the data. For these reasons, we argue that our data sample and this research provide a relevant representation of the population.

### Practical Implications

Although more and more medical applications embed artificial intelligence–based algorithms or agents, it is key for software developers to consider the physicians’ low level of familiarity toward artificial intelligence. When a radiologist asks a colleague his or her opinion about a patient’s x-ray, for instance, it is assumed that both went to medical schools and are physicians and had gone through a specific radiology training, regardless of where they come from. However, when the colleague is an artificial intelligence algorithm, things change drastically. In order for physicians to leverage the use of artificial intelligence–based applications, we argue that software developers should consider the following elements:

- Provide general information on how the artificial intelligence–based algorithm or software was built. Some topics would include information about the process as well as the types of data used and the amount of data used during the training and testing phases. It will allow physicians to gain understanding and trust.
- Integrate different user (physician) profiles with a dynamic level of guidance, according to the level of familiarity toward artificial intelligence. A physician with a low level of familiarity will require more information about the process by which the software treats the data. In contrast, a physician who is familiar with the topic only requires key information such as the confidence level.
- Describe the path that has led to each outcome or decision along with the level of confidence. It will allow the physician to understand the reasoning and the extent to which the outcome can supports his or her decision.
- Let the physician take the final decision, although the software provides the impact of this decision from a medical perspective. The documentation of the decision will then be used to improve the algorithm’s accuracy.

### Conclusions

On the basis of the number of current clinical trials leveraging artificial intelligence [36], the question is not whether artificial intelligence will be implemented in clinical settings but rather when it will become a standard in health care optimization. In the near future, practicing physicians will need to be equipped with the appropriate knowledge and skills to determine whether the artificial intelligence–based suggested diagnosis or treatment is appropriate. Thus, it is critical that physicians have a good understanding of the key concepts behind artificial intelligence. We believe that changes should first come from medical schools that should integrate AIM into their curriculum to both explain the origins and fundamentals of AIM and integrate AIM research throughout clinical topics from pathology to surgery, internal medicine, emergency medicine, and psychiatry, to name a few. By examining the individual components of AIM, our study informs existing research that highlights the needs to define what AIM content should be taught in undergraduate medical education [37]. This, in turn, requires university faculty to train and adapt their teaching material to this dynamic paradigm. By educating the physicians of tomorrow, they will act as drivers of change in their future placements.

At the same time, hospitals and clinics must emphasize on the importance of AIM and provide mandatory training for their medical professionals by means of continuing medical education or continuing professional development. To standardize and encourage both medical schools and hospitals to train their (future) physicians, governments can also play a key role by providing clear regulations, guidelines, and resources. Some countries such as the United Arab Emirates have already implemented national programs [38] to help all sectors integrate and regulate artificial intelligence. Therefore, we foresee future research focusing on assessing the outcomes of existing interventions (eg, lectures, modules, and training programs) in view of supporting medical schools, hospitals, and governments with the implementation of educational programs toward equipping medical professionals with relevant artificial intelligence skills.

## Appendix

Multimedia Appendix 1 Existing questionnaire-based research.

Multimedia Appendix 2 Questionnaire.

Multimedia Appendix 3 CHERRIES (Checklist for Reporting the Results of Internet E-Surveys) checklist.

Multimedia Appendix 4 Institutions participating.

## Abbreviations

AIM: artificial intelligence in medicine
KMO: Kaiser-Meyer-Olkin
MD: medical doctor
MS: medical student
