# Supplementary material for: Readiness to Embrace Artificial Intelligence Among Medical Doctors and Students: Questionnaire-Based Study
Source: JMIR Med Educ. 2022 Apr 12;8(2):e34973. doi: 10.2196/34973 (PMC9044144; doi:10.2196/34973)
Supplement: Multimedia Appendix 1 [file mededu_v8i2e34973_app1.docx]

### Multimedia Appendix 1. Existing questionnaire-based research.

| Authors | Year | Scope | Participants |
| --- | --- | --- | --- |
| [8] | 2021 | evaluate medical students’ perceptions about radiology and other medical specialties in relation to AI | 156 US-based medical students |
| [1] | 2021 | Explore sources of information about AI, AI applications and concerns, AI status as a topic in medicine, and students' feelings and attitudes | 3,133 medical and clinical students from around the world |
| [2] | 2021 | Analyze medical students' perceptions of the impact of artificial intelligence in radiology | 341 (could not access the article to collect more information) |
| [5] | 2020 | Assess the perceived impact of AI in radiology | 100 UK-based medical students |
| [3] | 2020 | Perceptions of AI among healthcare professionals | 98 healthcare professionals from the National Health Service in the UK |
| [6] | 2020 | How French health professionals perceive the arrival of AI in daily practice and the perception of the other actors involved in AI | 40 French-based physicians |
| [9] | 2019 | Investigate the benefits of AIM | 68 doctors, medical students, dental students, dentists, and students from around the world |
| [7] | 2019 | Investigate the awareness of AI assess physicians’ attitudes toward the medical application of AI | 669 Korean medical students and doctors |
| [4] | 2018 | Assess undergraduate medical students’ attitudes towards AI in radiology and medicine | 263 Germany-based medical students |

[1] Sotirios Bisdas, Constantin-Cristian Topriceanu, Zosia Zakrzewska, Alexandra-Valentina Irimia, Loizos Shakallis, Jithu Subhash, Maria-Madalina Casapu, Jose Leon-Rojas, Daniel Pinto dos Santos, Dilys Miriam Andrews, Claudia Zeicu, Ahmad Mohammad Bouhuwaish, Avinindita Nura Lestari, Lua’i Abu-Ismail, Arsal Subbah Sadiq, Almu’atasim Khamees, Khaled M. G. Mohammed, Estelle Williams, Aya Ibrahim Omran, Dima Y. Abu Ismail, and Esraa Hasan Ebrahim. 2021. Artificial Intelligence in Medicine: A Multinational Multi-Center Survey on the Medical and Dental Students’ Perception. *Front Public Health* 9, (December 2021), 795284. DOI:https://doi.org/10.3389/fpubh.2021.795284

[2] G. Caparrós Galán and F. Sendra Portero. 2021. Medical students’ perceptions of the impact of artificial intelligence in radiology. *Radiologia (Engl Ed)* (April 2021), S0033-8338(21)00084–9. DOI:https://doi.org/10.1016/j.rx.2021.03.006

[3] Simone Castagno and Mohamed Khalifa. 2020. Perceptions of Artificial Intelligence Among Healthcare Staff: A Qualitative Survey Study. *Frontiers in Artificial Intelligence* 3, (2020). Retrieved January 18, 2022 from https://www.frontiersin.org/article/10.3389/frai.2020.578983

[4] D. Pinto Dos Santos, Daniel Giese, S. Brodehl, S. H. Chon, W. Staab, R. Kleinert, D. Maintz, and B. Baeßler. 2019. Medical students’ attitude towards artificial intelligence: a multicentre survey. *European radiology* 29, 4 (2019), 1640–1646.

[5] P. Kasetti and R. Botchu. 2020. The Impact of Artificial Intelligence in Radiology: As Perceived by Medical Students. *Russian Electronic Journal of Radiology* 10, 4 (2020), 179–185.

[6] M.-C. Laï, M. Brian, and M.-F. Mamzer. 2020. Perceptions of artificial intelligence in healthcare: findings from a qualitative survey study among actors in France. *Journal of Translational Medicine* 18, 1 (2020), 1–13.

[7] Songhee Oh, Jae Heon Kim, Sung-Woo Choi, Hee Jeong Lee, Jungrak Hong, and Soon Hyo Kwon. 2019. Physician confidence in artificial intelligence: An online mobile survey. *Journal of medical Internet research* 21, 3 (2019), e12422.

[8] Christian J. Park, Paul H. Yi, and Eliot L. Siegel. 2021. Medical Student Perspectives on the Impact of Artificial Intelligence on the Practice of Medicine. *Current Problems in Diagnostic Radiology* 50, 5 (September 2021), 614–619. DOI:https://doi.org/10.1067/j.cpradiol.2020.06.011

[9] Samira Yeasmin. 2019. Benefits of Artificial Intelligence in Medicine. In *2019 2nd International Conference on Computer Applications & Information Security (ICCAIS)*, IEEE, 1–6.
