# Supplementary material for: Readiness to Embrace Artificial Intelligence Among Medical Doctors and Students: Questionnaire-Based Study
Source: JMIR Med Educ. 2022 Apr 12;8(2):e34973. doi: 10.2196/34973 (PMC9044144; doi:10.2196/34973)
Supplement: Multimedia Appendix 2 [file mededu_v8i2e34973_app2.docx]

### Multimedia Appendix 2. Questionnaire.

What is your gender?

- Female
- Male
- Prefer not to say

What is your age group?

- <20
- 20-29
- 30-39
- 40-49
- 50-59
- 60-69
- >70

Where are you based?

- Asia
- Africa
- Central America
- South America
- Europe
- Eastern Europe
- Middle East
- Oceania

You are a?

- Medical student
- Medical doctor

What is your specialization? (only available to medical doctors)

- List of 18 specializations + Other with a text field

From which university did you (or will you) obtain your highest medical degree (e.g., MBBS, MD, DO)?

- Text field

Where did you (or from where will you) obtain your highest medical degree (e.g., MBBS, MD, DO)?

- Asia
- Africa
- Central America
- South America
- Europe
- Eastern Europe
- Middle East
- Oceania

When did you (or will you) obtain your professional medical degree (i.e., highest professional degree)?

- Text field

How familiar are you with…

|  | Never heard of it | Heard of it few times | Understand it | Can potentially explain it | Can confidently explain it |
| --- | --- | --- | --- | --- | --- |
| Artificial intelligence | O | O | O | O | O |
| Machine Learning (ML) | O | O | O | O | O |
| Supervised ML | O | O | O | O | O |
| Unsupervised ML | O | O | O | O | O |
| Deep Learning | O | O | O | O | O |
| Natural network(s) | O | O | O | O | O |
| Fuzzy logic | O | O | O | O | O |
| Support vector machine | O | O | O | O | O |
| Overfitting/underfitting | O | O | O | O | O |
| Feature selection | O | O | O | O | O |

Have you attended a course on artificial intelligence?

- Never
- This year
- Last year
- Two to three years ago
- More than three years ago

Would you benefit from more training to:

|  | Strongly disagree | Disagree | Neutral | Agree | Strongly agree |
| --- | --- | --- | --- | --- | --- |
| Better understand the main concept of AI | O | O | O | O | O |
| Explore new opportunities offered by AI in general | O | O | O | O | O |
| Explore new opportunities offered by AI in medicine and in my field | O | O | O | O | O |
| Know more of existing commercial solutions | O | O | O | O | O |
| Create my own AI algorithms or applications | O | O | O | O | O |

In the context of healthcare, who else would benefit from more training and educational programs on AIM?

|  | Strongly disagree | Disagree | Neutral | Agree | Strongly agree |
| --- | --- | --- | --- | --- | --- |
| Medical students | O | O | O | O | O |
| Residents | O | O | O | O | O |
| Practicing physicians | O | O | O | O | O |
| General public / patients | O | O | O | O | O |
| Hospital administrators | O | O | O | O | O |
| Policymakers | O | O | O | O | O |
| Caregivers | O | O | O | O | O |

In your field, which of the following issues are important for the development and implementation of AIM?

|  | Strongly disagree | Disagree | Neutral | Agree | Strongly agree | I do not know |
| --- | --- | --- | --- | --- | --- | --- |
| Outcomes of AI algorithms are difficult to trace or understand (the blackbox syndrome) | O | O | O | O | O | O |
| The complexity of the field of medicine | O | O | O | O | O | O |
| The availability of high-quality data samples | O | O | O | O | O | O |
| The AI's level of autonomy (what AI should and should not do) | O | O | O | O | O | O |
| The costs associated with the implementation of AI | O | O | O | O | O | O |
| Data privacy / confidentiality | O | O | O | O | O | O |

In your field, which factors are the most important for driving the implementation of AIM?

|  | Strongly disagree | Disagree | Neutral | Agree | Strongly agree | I do not know |
| --- | --- | --- | --- | --- | --- | --- |
| The availability of comparison studies | O | O | O | O | O | O |
| The safe use of AI | O | O | O | O | O | O |
| Build trust between Humans and AI | O | O | O | O | O | O |
| Availability of regulations and legislations | O | O | O | O | O | O |
| The top management's level of understanding | O | O | O | O | O | O |

What do you consider the risks of AIM?

|  | Strongly disagree | Disagree | Neutral | Agree | Strongly agree |
| --- | --- | --- | --- | --- | --- |
| Dehumanization of healthcare | O | O | O | O | O |
| Reduction in physicians' skills (e.g., physicians might execute fewer types of tasks) | O | O | O | O | O |
| AI will eventually harm patients | O | O | O | O | O |
| Physicians may become redundant | O | O | O | O | O |

Could you see yourself one day working with an AI algorithm as if it were your colleague?

- Yes
- No

Could you tell us why you answered that way in the question above?

- Text field

Thank you very much for your time. Please add any comment

- Text field
