# Supplementary material for: Readiness to Embrace Artificial Intelligence Among Medical Doctors and Students: Questionnaire-Based Study
Source: JMIR Med Educ. 2022 Apr 12;8(2):e34973. doi: 10.2196/34973 (PMC9044144; doi:10.2196/34973)
Supplement: Multimedia Appendix 4 [file mededu_v8i2e34973_app4.docx]

### Multimedia Appendix 4. Institutions participating.

| Type | University | Region | Graduation year | Number of participants |
| --- | --- | --- | --- | --- |
| MD | Aden University | Middle East | 1990 | 1 |
| MD | Aden University | Middle East | 2010 | 1 |
| MD | Ain Shams University | Africa | 1986 | 1 |
| MD | Alfaisal University | Middle East | 2017 | 1 |
| MD | All India Institute of Medical Sciences | Asia | 2019 | 1 |
| MD | Antonius Ziekenhuis Nieuwegein Nederland | Europe | 1988 | 1 |
| MD | The Arab Board Of Health Specializations | Middle East | 2014 | 1 |
| MS | Aston University | Europe | 2024 | 1 |
| MS | American University of Beirut | Middle East | 2021 | 2 |
| MD | B.N Mandal University | Asia | 2006 | 1 |
| MD | Baghdad Medical College | Middle East | 1986 | 1 |
| MD | Bangalore Medical College | Asia | 1986 | 1 |
| MD | Bangalore Medical College | Asia | 2020 | 1 |
| MS | Barts and the London | Europe | 2021 | 1 |
| MD | Baylor University | North America | 1990 | 1 |
| MS | Bezmialem Vakif University | Eastern Europe | 2021 | 2 |
| MS | Birmingham University | Europe | 2022 | 2 |
| MD | Bombay University | Asia | 1984 | 1 |
| MS | Brighton and Sussex Medical School | Europe | 2022 | 1 |
| MD | Bristol | Europe | 2007 | 1 |
| MD | Bristol | Europe | 2020 | 1 |
| MD | Bukovinian State Medical University | Eastern Europe | 2016 | 1 |
| MD | Cairo University | Africa | 2017 | 1 |
| MD | Cambridge University | Europe | 2018 | 2 |
| MS | Cambridge University | Europe | 2022 | 1 |
| MD | Charles Universioty Prague | Europe | 1984 | 1 |
| MD | Dow Medical College | Asia | 2000 | 1 |
| MD | TN MGR medical university | Asia | 1999 | 1 |
| MD | South Africa College of Medicine | Africa | 2003 | 1 |
| MD | Devi Ahilya Vishwavidyalaya | Asia | 2003 | 1 |
| MD | Dayanand Medical College | Asia | 2019 | 1 |
| MS | Debrecen University | Europe | 2022 | 1 |
| MD | Dharwad University | Asia | 1994 | 1 |
| MD | Dubai Medical College | Middle East | 2002 | 1 |
| MD | Dubai Medical College | Middle East | 2003 | 1 |
| MD | Dubai Medical College | Middle East | 2019 | 1 |
| MS | Dubai Medical College | Middle East | 2022 | 3 |
| MD | Mayo Medical School | North America | 1982 | 1 |
| MD | DR. N.T.R University of Health Sciences | Asia | 2020 | 1 |
| MS | Dubai Medical College | Middle East | 2021 | 1 |
| MS | Dubai Medical College | Middle East | 2023 | 1 |
| MD | Dubai Medical College | Middle East | 2003 | 1 |
| MD | University of Edinburgh | Europe | 2012 | 1 |
| MD | University of Edinburgh | Europe | 1985 | 1 |
| MD | Cairo University | Africa | 2010 | 1 |
| MD | Emory University | North America | 2005 | 1 |
| MS | GMERS Medical Collage | Asia | 2022 | 1 |
| MD | Government Medical College, Thrissur | Asia | 2007 | 1 |
| MS | Gujarat University | Asia | 2025 | 1 |
| MD | Gulf Medical University | Middle East | 2016 | 1 |
| MS | Hassan II University of Casablanca | Africa | 2027 | 1 |
| MS | Indira Gandhi Government Medical College & Hospital | Asia | 2023 | 1 |
| MS | Jinnah Medical and Dental College | Asia | 2021 | 2 |
| MS | Kabul University of Medical Sciences | Middle East | 2022 | 2 |
| MS | Kaloji Narayan Rao University of Health Sciences | Asia | 2023 | 1 |
| MS | Keck School of Medicine of USC | North America | 2023 | 2 |
| MD | Kerala University | Asia | 1995 | 1 |
| MD | Kharkiv National Medical University | Europe | 2020 | 2 |
| MD | University of Khartoum | Middle East | 2003 | 1 |
| MD | King Abdulaziz University | Middle East | 2014 | 1 |
| MD | King Edward medical University | Asia | 2001 | 1 |
| MD | King Edward medical University | Asia | 2004 | 1 |
| MD | King Edward medical University | Asia | 2009 | 1 |
| MD | King Edward medical University | Asia | 2015 | 1 |
| MS | King George's Medical University Lucknow | Asia | 2023 | 2 |
| MD | King's College London | Europe | 1988 | 1 |
| MD | King's College London | Europe | 2013 | 1 |
| MS | King's College London | Europe | 2014 | 1 |
| MS | KLE Deemed University | Asia | 2021 | 1 |
| MD | KU Leuven | Europe | 2014 | 1 |
| MD | Lebanese University | Middle East | 2017 | 2 |
| MS | LECOM | North America | 2022 | 2 |
| MS | Liverpool University | Europe | 2023 | 2 |
| MD | Madras University | Asia | 1987 | 1 |
| MS | Manipal Academy of Higher Education | Asia | 2021 | 2 |
| MD | Manipal Academy of Higher Education | Asia | 2020 | 1 |
| MS | Marmara university | Europe | 2021 | 1 |
| MD | Marmara university | North America | 1982 | 1 |
| MS | University of Lancashire | Europe | 2023 | 1 |
| MS | Mohammed Bin Rashid University of Medicine | Middle East | 2025 | 3 |
| MS | Mohammed Bin Rashid University of Medicine | Middle East | 2026 | 3 |
| MS | Mohammed Bin Rashid University of Medicine | Middle East | 2024 | 3 |
| MS | Mohammed Bin Rashid University of Medicine | Middle East | 2023 | 3 |
| MS | Mohammed Bin Rashid University of Medicine | Middle East | 2022 | 2 |
| MS | McMaster University | North America | 2022 | 1 |
| MD | Universidad Juárez del Estado de Durango | North America | 1994 | 1 |
| MS | Universidad de Monterrey | North America | 2019 | 1 |
| MD | Medical University of Warsaw | Europe | 2018 | 1 |
| MD | MGR Medical University | Asia | 1999 | 1 |
| MD | MGR Medical University | Asia | 2000 | 1 |
| MD | Mosul Medical College | Middle East | 1986 | 1 |
| MD | University of Mumbai | Asia | 1994 | 1 |
| MD | University of Nagpur | Asia | 1994 | 1 |
| MS | Newcastle University | Europe | 2021 | 1 |
| MD | Newport University California | North America | 1992 | 1 |
| MD | University of Otago | Oceania | 1988 | 1 |
| MD | Oxford University | Europe | 1985 | 1 |
| MD | Pune University | Asia | 1986 | 1 |
| MD | Punjab University | Asia | 1987 | 1 |
| MS | Queen’s University Belfast | Europe | 2023 | 1 |
| MD | Rajiv Gandhi University | Asia | 2012 | 1 |
| MS | Rajiv Gandhi University | Asia | 2023 | 1 |
| MD | RAK Medical & Health Sciences University | Middle East | 2020 | 1 |
| MS | RAK Medical & Health Sciences University | Middle East | 2025 | 2 |
| MS | RAK Medical & Health Sciences University | Middle East | 2026 | 1 |
| MS | Royal College of Surgeons Ireland – Barhain | Middle East | 2023 | 1 |
| MS | Royal College of Surgeons Ireland – Barhain | Middle East | 2025 | 1 |
| MS | Royal College of Surgeons Ireland – Barhain | Middle East | 2022 | 1 |
| MS | Riga Stradins University | Europe | 2024 | 1 |
| MD | Royal College of Obstetricians and Gynaecologists | Europe | 2002 | 1 |
| MD | Royal College of Paediatrics and Child Health | Europe | 2009 | 1 |
| MD | Royal College of Surgeons Ireland | Europe | 2014 | 1 |
| MD | Royal College of Surgeons Ireland | Europe | 2018 | 1 |
| MD | Royal College Physicians and Surgeons Glasgow | Europe | 2012 | 1 |
| MD | Sanaa University | Middle East | 2008 | 1 |
| MD | Saurashtra University | Asia | 1994 | 1 |
| MD | Semmelweis University of Medical Siences | Europe | 1995 | 1 |
| MD | Seoul National University College of Medicine | Asia | 1998 | 1 |
| MD | University of Southampton | Europe | 1998 | 1 |
| MD | University of Southampton | Europe | 2003 | 1 |
| MD | St. George's University | North America | 2004 | 1 |
| MD | Stellenbosch University | Africa | 2016 | 1 |
| MS | The Aga Khan University Hospital | Asia | 2021 | 1 |
| MS | The University of Adelaide | Oceania | 2021 | 1 |
| MD | TN MGR Medical University | Asia | 2004 | 1 |
| MD | Tripoli University | Europe | 2005 | 1 |
| MD | University of the United Arab Emirates | Middle East | 1997 | 1 |
| MD | University of the United Arab Emirates | Middle East | 2007 | 1 |
| MD | University of the United Arab Emirates | Middle East | 2005 | 1 |
| MS | University of the United Arab Emirates | Middle East | 2022 | 2 |
| MS | University of the United Arab Emirates | Middle East | 2024 | 1 |
| MS | University of the United Arab Emirates | Middle East | 2026 | 1 |
| MD | University College London | Europe | 2013 | 1 |
| MS | University College London | Europe | 2022 | 1 |
| MD | University of Montreal | North America | 2017 | 1 |
| MS | University of Montreal | North America | 2025 | 1 |
| MD | Universidad de Monterrey | North America | 2003 | 1 |
| MD | Universidad de Murcia | Europe | 2012 | 1 |
| MD | Universidad Veracruzana | South America | 2012 | 1 |
| MS | Universitas Indonesia | Asia | 2025 | 1 |
| MS | Universitas Indonesia | Asia | 2024 | 1 |
| MS | Universitas Indonesia | Asia | 2023 | 1 |
| MS | University College Dublin | Europe | 2024 | 1 |
| MD | University College London | Europe | 2001 | 1 |
| MS | University College of Medicine and Dentistry | Asia | 2021 | 1 |
| MS | University of Birmingham | Europe | 2023 | 1 |
| MD | University of Birmingham - Alabama | North America | 2004 | 1 |
| MS | University of Bristol | Europe | 2022 | 1 |
| MD | University of Cambridge | Europe | 1988 | 1 |
| MS | University of Crete | Europe | 2021 | 1 |
| MS | University of Debrecen | Europe | 2024 | 1 |
| MS | University of Ibadan | Africa | 2023 | 1 |
| MS | University of Ibadan | Africa | 2021 | 1 |
| MD | University of Kerala | Asia | 2015 | 1 |
| MD | University of Kerala | Asia | 1997 | 1 |
| MS | University of Leicester | Europe | 2024 | 1 |
| MS | University of Manchester | Europe | 2022 | 1 |
| MS | University of Miami | North America | 2021 | 1 |
| MD | University of Munster | Europe | 1994 | 1 |
| MS | University of Notre Dame | Oceania | 2023 | 1 |
| MD | University of Ottawa | North America | 2008 | 1 |
| MD | University of Ottawa | North America | 2016 | 1 |
| MS | University of Sharjah | Middle East | 2022 | 1 |
| MS | University of Sharjah | Middle East | 2023 | 3 |
| MS | University of Sharjah | Middle East | 2026 | 1 |
| MD | University of Southern California Keck School of Medicine | North America | 1998 | 1 |
| MD | University of Stellenbosch | Africa | 1998 | 1 |
| MD | University of Wales | Europe | 1988 | 1 |
| MD | University of Kentucky | North America | 1990 | 1 |
| MS | University of Pittsburg | North America | 2023 | 1 |
| MS | University of Pittsburg | North America | 2024 | 1 |
| MS | Vydehi Medical College | Asia | 2022 | 1 |
| MS | Weill Cornell Medicine - Qatar | Middle East | 2022 | 3 |
| MD | West Bengal University of Health Sciences | Asia | 2020 | 1 |
| MS | Yale University | North America | 2024 | 1 |
| MS | Ziauddin University | Asia | 2023 | 1 |
